# Inactivation of tomato *WAT1* leads to reduced susceptibility to *Clavibacter michiganensis* through downregulation of bacterial virulence factors

**DOI:** 10.3389/fpls.2023.1082094

**Published:** 2023-05-31

**Authors:** Eleni Koseoglou, Katharina Hanika, Mas M. Mohd Nadzir, Wouter Kohlen, Jan M. van der Wolf, Richard G. F. Visser, Yuling Bai

**Affiliations:** ^1^ Plant Breeding, Wageningen University & Research, Wageningen, Netherlands; ^2^ Graduate School Experimental Plant Sciences Wageningen University & Research, Wageningen, Netherlands; ^3^ Cluster of Plant Developmental Biology, Laboratory of Molecular Biology, Wageningen University & Research, Wageningen, Netherlands; ^4^ Biointeractions & Plant Health, Wageningen University & Research, Wageningen, Netherlands

**Keywords:** susceptibility genes, *Clavibacter michiganensis*, CRISPR/Cas9, auxin, ethylene, disease, Walls Are Thin1, bacterium

## Abstract

Tomato bacterial canker caused by *Clavibacter michiganensis* (Cm) is considered to be one of the most destructive bacterial diseases of tomato. To date, no resistance to the pathogen has been identified. While several molecular studies have identified (*Cm*) bacterial factors involved in disease development, the plant genes and mechanisms associated with susceptibility of tomato to the bacterium remain largely unknown. Here, we show for the first time that tomato gene *SlWAT1* is a susceptibility gene to *Cm*. We inactivated the gene *SlWAT1* through RNAi and CRISPR/Cas9 to study changes in tomato susceptibility to *Cm*. Furthermore, we analysed the role of the gene in the molecular interaction with the pathogen. Our findings demonstrate that *SlWAT1* functions as an S gene to genetically diverse *Cm* strains. Inactivation of *SlWAT1* reduced free auxin contents and ethylene synthesis in tomato stems and suppressed the expression of specific bacterial virulence factors. However, CRISPR/Cas9 *slwat1* mutants exhibited severe growth defects. The observed reduced susceptibility is possibly a result of downregulation of bacterial virulence factors and reduced auxin contents in transgenic plants. This shows that inactivation of an S gene may affect the expression of bacterial virulence factors.

## Introduction

Plant disease resistance is genetically controlled, mostly by dominantly inherited, race specific resistance (R) genes. In the presence of corresponding pathogen-derived effectors many R genes confer resistance through effector-triggered immunity (ETI) (Jones and Dangl, 2006). In plant-microbe interactions, resistance is a common outcome. In fact, a high degree of adaptation is required for microbes to become pathogenic (Hückelhoven et al., 2013). During their co-evolution pathogens have found ways to target and manipulate plant genes, referred to as susceptibility (S) genes, to promote disease development (Bai et al., 2008; Pavan et al., 2011; Appiano et al., 2015; Pessina et al., 2016; Acevedo-Garcia et al., 2017; Berg et al., 2017). S genes are important for biological functions of plants, which appears to be a significant factor in their retainment across species (Hückelhoven et al., 2013). This is exemplified by the *Mildew Locus O* (*MLO*) gene family that has been identified in plant species such as barley, tomato, Arabidopsis, grape, apple and cucumber (Cohn et al., 2014; Cox et al., 2017; Oliva et al., 2019). In contrast to dominant R genes, loss-of-function of S genes can potentially lead to recessively inherited, broad-spectrum, and durable resistance (van Damme et al., 2008; Pavan et al., 2010; Pavan et al., 2011; Huibers et al., 2013; Sun et al., 2016a; Berg et al., 2017; Santillán Martínez et al., 2020). For example, loss-of-function of genes in the glutamate decarboxylases (GADs) family provide enhanced resistance against the vascular bacterium *Ralstonia solanacearum* in Arabidopsis and tomato (*Solanum lycopersicum*) (Xian et al., 2020). In addition, mutation of *Sugars Will Eventually Be Exported Transporter* (*SWEET)* genes in multiple plant species has been demonstrated to be an effective strategy to obtain resistance to *Xanthomonas* spp (Cohn et al., 2014; Cox et al., 2017; Oliva et al., 2019).

Bacterial canker of tomato caused by the Gram-positive bacterium *Clavibacter michiganensis* (*Cm*), is considered to be one of the most important seed-borne diseases of tomato worldwide (Davis et al., 1984; Gartemann et al., 2008; Nouioui et al., 2018). The pathogen colonizes the vasculature of plants leading to systemic infections that result in wilting of leaves, vascular tissue necrosis and formation of cankers on the stems and petioles of plants, eventually leading to plant death (Eichenlaub and Gartemann, 2011; Sen et al., 2015; Chalupowicz et al., 2017).

On the molecular level of the tomato-*Cm* interaction, several bacterial factors involved in virulence are known. Full virulence of the *Cm* reference strain NCPBB382 requires the presence of two native plasmids, pCM1 and pCM2, where the major virulence factors *celA* and *pat-1* are located (Gartemann et al., 2008; Savidor et al., 2014). Loss of either of the plasmids leads to reduced virulence and loss of both results in an endophytic nonvirulent strain (Meletzus et al., 1993; Savidor et al., 2012). Several other proteins are encoded by genes located on the circular chromosome of *Cm* that are involved in the colonization of plants and induction of disease symptoms (Gartemann et al., 2008; Savidor et al., 2012; Savidor et al., 2014; Thapa et al., 2017). Such genes, include the transcriptional factors *vatr1* and *vatr2*, which act in the regulation of several other virulence genes. *vatr1* and *vatr2* are involved in the regulation of virulence factors, such as the endo- beta- 1,4- glucanase *celA*, subtilase proteinase *SbtC* and the serine proteases *pat-1* and *PhpA*, both on the chromosome and plasmids of *Cm* (Savidor et al., 2014).

While several molecular studies have identified bacterial factors involved in disease development, the mechanisms associated with susceptibility of tomato to the bacterium remain largely unknown. The only experimentally confirmed plant factor involved in disease development is the phytohormone ethylene (Balaji et al., 2008; Savidor et al., 2012; Savidor et al., 2014). During infection *Cm* promotes the production of host-derived ethylene by specifically upregulating the ethylene biosynthetic gene *ACO1*. Mutant *Never ripe* (*Nr*) tomato plants with impaired ethylene perception display significant *Cm* symptom development delay (Balaji et al., 2008). The observation that the nonvirulent *Cmm100* strain lacks the ability to induce the production of host derived ethylene further highlights the importance of ethylene in *Cm* symptom development (Savidor et al., 2012).

Despite extensive screenings of wild germplasm, resistance to the pathogen has not been identified yet (Sen et al., 2015). In our study, we hypothesized that impairment of S genes involved in the tomato-*Cm* interaction might result in loss-of-susceptibility. Therefore, we set out to identify tomato S genes potentially involved in the interaction.

Recently, the tomato ortholog of *Walls Are Thin1* (*SlWAT1*) was identified and inactivated through RNAi and CRISPR/Cas9 (Hanika et al., 2026). CRISPR/Cas9 mediated knock-out of the gene led to resistance to the vascular fungi *Verticillium dahliae*, *V. albo-altrum* and *Fusarium oxysporum f.* sp. *lycopersici* (Hanika et al., 2026). The Arabidopsis *WAT1* gene encodes for a tonoplast localized plant-specific protein. *WAT1* has been shown to be involved in vacuolar auxin transport and secondary cell wall biosynthesis (Ranocha et al., 2010; Ranocha et al., 2013). In *Arabidopsis* loss-of-function of the gene leads to enhanced resistance to a broad range of vascular pathogens, including the bacterium *Ralstonia solanacearum* (Denance et al., 2013). In cotton (*Gosypium hirsutum*) three *WAT* homologs have been identified. Simultaneous transient silencing of the cotton genes enhanced resistance to the vascular fungus *V. dahliae* (Tang et al., 2019). In both cotton and Arabidopsis resistance involves the repression of indole metabolism and altered contents of indole-3-acetic acid (IAA) and salicylic acid (SA) (Denance et al., 2013; Tang et al., 2019). In addition, local lignin deposition was associated with *V. dahliae* resistance in cotton (Tang et al., 2019). In this study, we show that impairment of *SlWAT1* through RNAi and CRISPR/Cas9 leads to broad-spectrum reduced susceptibility to genetically different *Cm* strains. Next to this, we show that downregulation of *SlWAT1* reduces auxin content in tomato stems and potentially affects the expression of bacterial virulence factors.

## Results

### Down-regulation of *SlWAT1* leads to broad-spectrum reduced susceptibility to *Cm*

**Figure 1:**
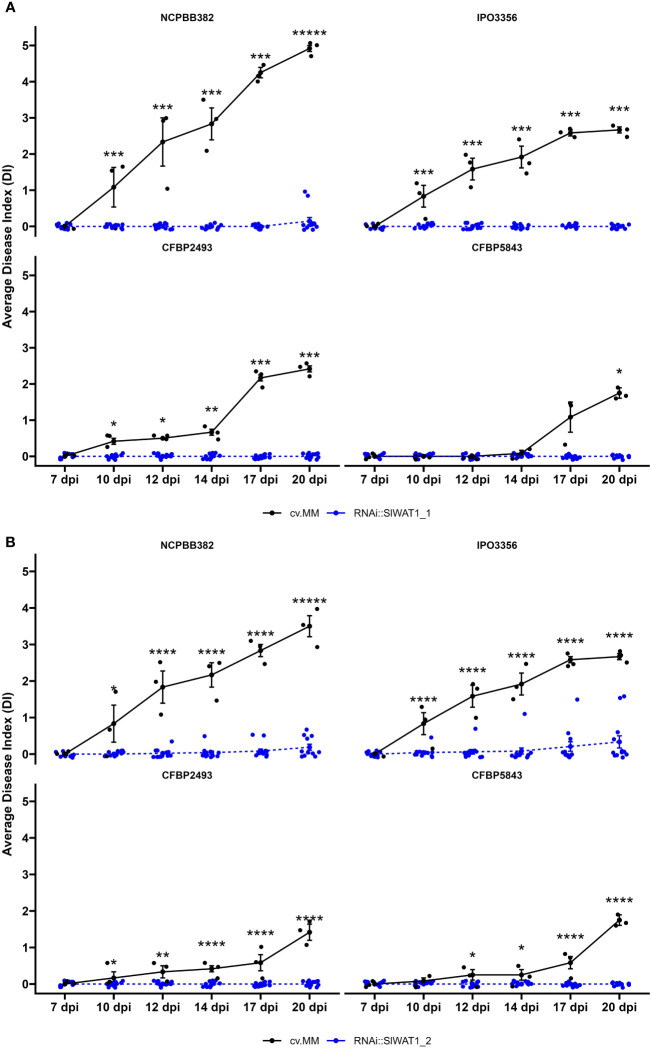
Disease index of *SlWAT1* RNAi silenced lines inoculated with genetically diverse *Cm* strains. Wilting symptom development of **(A)** RNAi::SlWAT1_1 and **(B)** RNAi::SlWAT1_2 lines compared to the background donor susceptible control cv. Moneymaker (cv.MM) from 7 days post inoculation (dpi) to 20 dpi. Means of both RNAi::SlWAT1 lines were significantly different from the cv.MM controls, for all strains used in the disease assay (n=12). Bars indicate the standard errors. Asterisks indicate significant differences (Student’s t-test, *p ≤ 0.05; ***p ≤ 0.001,****p ≤ 0.0001).

To study the role of *SlWAT1* in susceptibility of tomato to *Cm*, homozygous T_3_ progeny of two RNAi lines (RNAi::SlWAT1_1 (TV181036) and RNAi::SlWAT1_2 (TV181034)) derived from two independent transformants in cv. Moneymaker (cv. MM) background were used (Hanika et al., 2026). Expression analysis of the T_2_ parental lines, revealed that the relative residual expression of lines RNAi::SlWAT1_1 and RNAi::SlWAT1_2 was on average 20% and 54%, respectively (Hanika et al., 2026). To evaluate the spectrum of resistance conferred by silencing of *SlWAT1*, lines RNAi::SlWAT1_1 and RNAi::SlWAT1_2 were challenged with four genetically diverse *Cm* strains (Jacques et al., 2012). Wilting symptoms on the infected plants were recorded from 7 to 20 days post inoculation (dpi). Severe wilting symptoms caused by all four genetically distinct *Cm* stains were observed on MM plants. Aggressiveness of the four strains differed, with NCPBB382 being the most aggressive and CFBP5843 being the least aggressive strain (**Figure S1**). Both T_3_ lines used in the disease assays exhibited significant reduction of wilting symptoms to all tested strains (**Figure 1**). Mild wilting symptoms were observed on RNAi::SlWAT1_1 transgenic plants when inoculated with the most aggressive NCPBB382 strain. For RNAi::SlWAT1_2 mild symptoms were observed for strains NCPBB382 and IPO3356.

### Bacterial growth is not limited in RNAi::SlWAT1_1 transgenic plants

**Figure 2:**
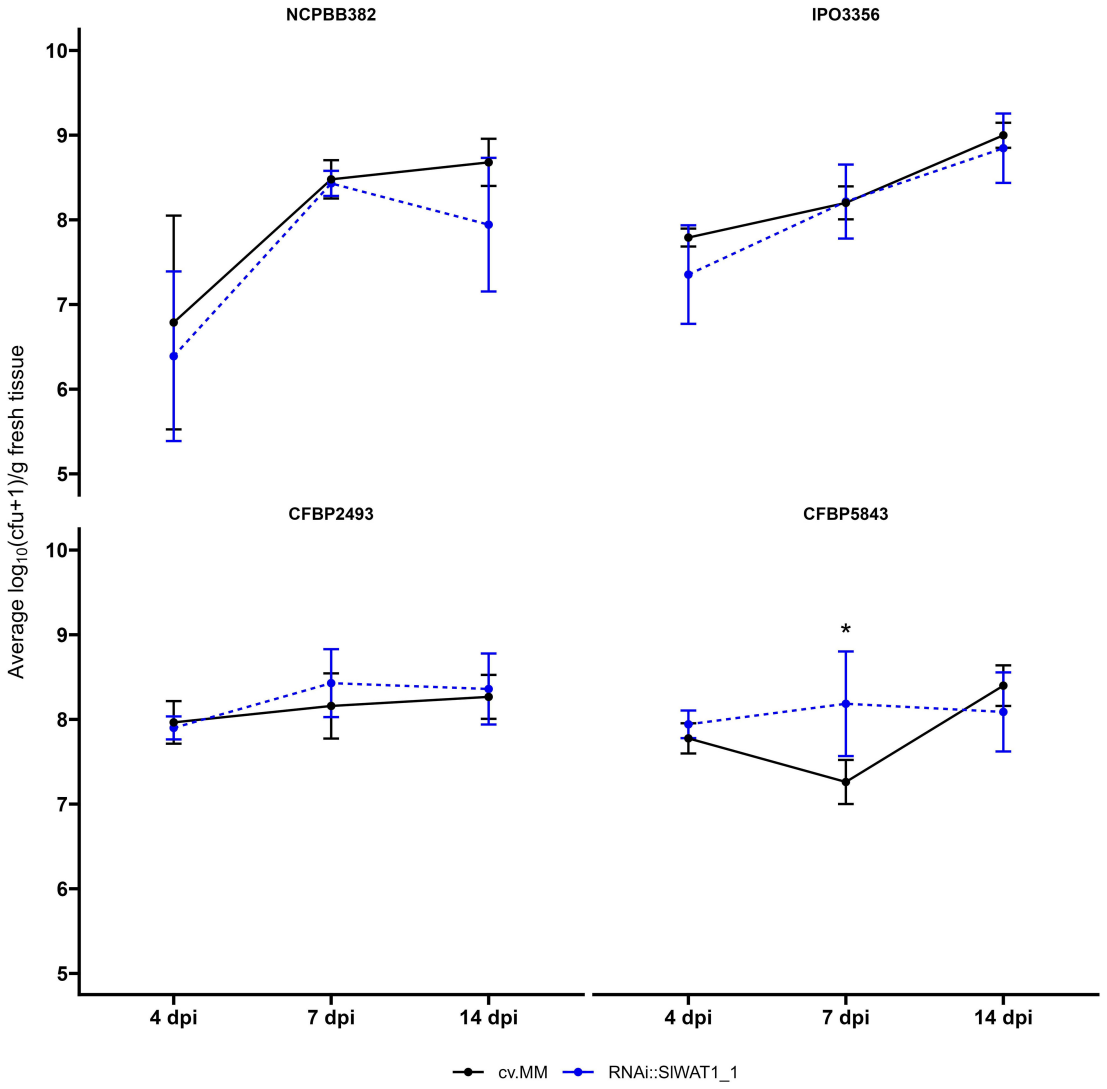
*Clavibacter michiganensis* population dynamics in cv. Moneymaker (cv.MM) and RNAi::SlWAT1_1 transgenic plants. Bacterial titers of the four bacterial strains used in the experiments were quantified at 4, 7 and 14 days post inoculation (dpi). Five biological replicates (n=5) were used per time point and bacterial strain. Lines represent the average log_10_(cfu+1/g fresh tissue) ± stdev. The experiments were repeated independently at least twice with similar results. Asterisks indicate statistical differences (Student’s t-test, *p ≤ 0.05).

To determine whether the reduction in observable wilting symptoms in the RNAi lines was correlated with changes in the bacterial growth *in planta*, the population dynamics of the four different strains were quantified at three time points (4 dpi, 7 dpi and 14 dpi). An estimated 5x10^5^ colony forming units (cfu)/mL was used for the inoculation of the plants. Over the course of infection, all *Cm* strains reached high population densities (~10^9^ log_10_(cfu+1/g fresh stem tissue)). No significant statistical differences in population densities were observed between the susceptible cv. MM and transgenic plants for strains NCPBB382, IPO3356, CFBP5843 and CFBPB2493 (**Figure 2**).

### CRISPR/Cas9- mediated knock-out of *SlWAT1* leads to loss-of-susceptibility to *Cm* without limiting bacterial growth

**Figure 3:**
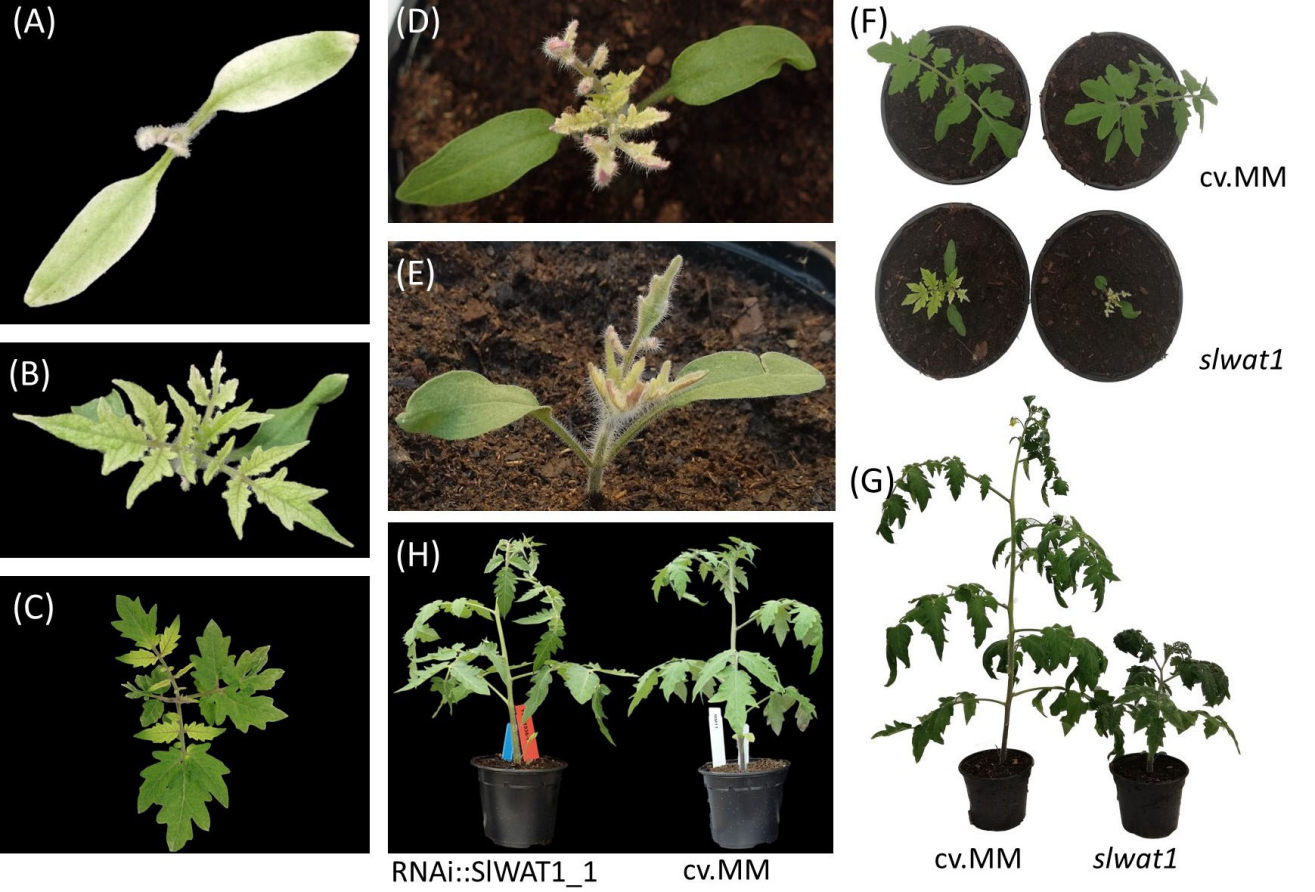
Pleiotropic phenotypes of *slwat1* knock-out mutant plants. **(A-C)** Lack of chlorophyll observed in developing *slwat1* mutants. **(D, E)** Anthocyanin accumulation in the abaxial side of leaves of developing mutants. Severe growth retardation in four weeks old **(F)** and ten weeks old **(G)** *slwat1* mutants compared to the cv. Moneymaker (cv.MM). **(H)** phenotype of 6 weeks old RNAi::SlWAT1_1 transgenic plants compared to cv.MM.

To exclude the possibility of interference of the residual expression of *SlWAT1* in the RNAi lines with the phenotype observed and to confirm our previous results, we decided to include a CRISPR/Cas9 mutant line in the experiments. Silencing of *SlWAT1* through RNAi did not lead to any observable adverse pleiotropic effects (**Figure 3H**). However, for the gene edited mutant line *slwat1*, severe growth retardation was observed, as previously described (**Figure 3**) (Hanika et al., 2026). Besides the severe growth retardation, lack of chlorophyll and strong accumulation of anthocyanins at the abaxial side of developing leaves at early developmental stages were also observed. The latter phenotypic abnormalities were alleviated as the plants grew older (**Figures 3C, G**).

**Figure 4:**
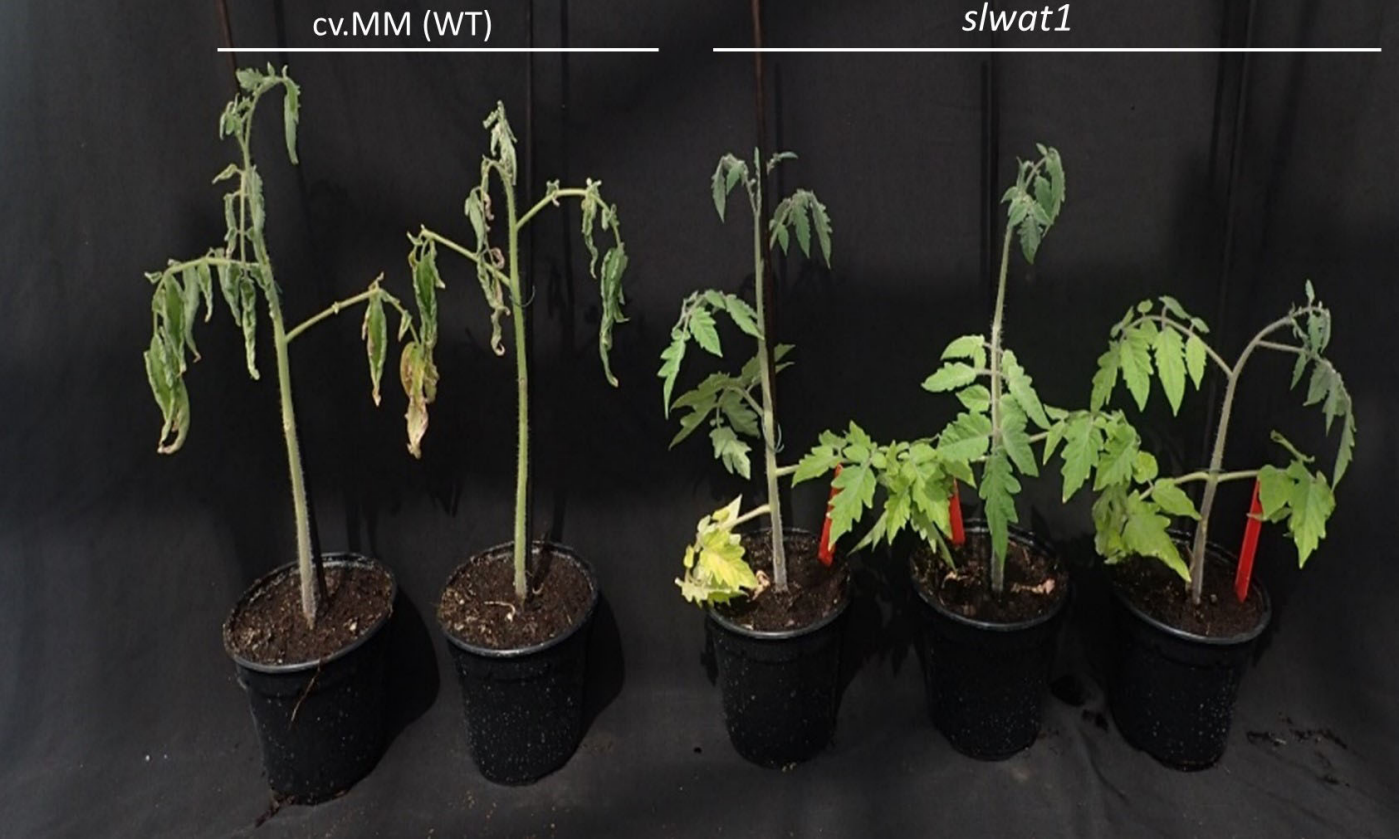
Symptom development of mutants *slwat1* in comparison to the susceptible background cv.MM inoculated with strain NCPBB382 at 20 days post inoculation.

Changes of tomato susceptibility in response to *Cm* due to different developmental stages have previously been reported. Generally, the severity of disease decreases and the incubation period becomes longer with inoculations at later developmental stages (Chang et al., 1992; Sharabani et al., 2013). Therefore, for the inoculation of the plants we decided to use control plants at the same developmental stage as the mutants (4^th^ leaf stage). To achieve synchroneity in the developmental stages of our two genotypes control plants were sown every week. When the control plants and the *slwat1* mutants were at the same developmental stage we challenged them with the hypervirulent strain NCPBB382. At 20 dpi, severe wilting symptoms were observed in the susceptible background cv. MM. No symptoms were observed in the *slwat1* mutants, confirming our previous results (**Figures 4**, **S2A**). When the *in planta* bacterial titers were quantified, no significant statistical changes were found between the susceptible cv. MM and *slwat1* mutants (**Figure S2B**).

### Silencing of *SlWAT1* reduces auxin content and affects the expression of auxin related genes

**Figure 5:**
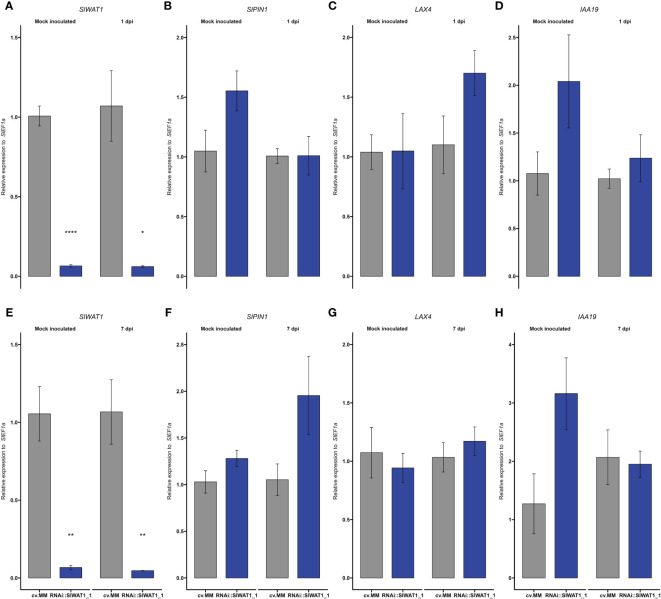
Expression of auxin transporter/signaling genes and auxin content is reprogrammed in RNAi::SlWAT1 plants. Relative expression of genes **(A, E)** *SlWAT1*, **(B, F)** *SlPIN1*, **(C, G)** *LAX4* and **(D, H)** *IAA19* in mock treated and *Cm* inoculated plants at 1 day post inoculation (dpi) and 7 dpi. Fold changes were normalized relative to expression of the *SlEf1α* in cv.MM plants. Bars represent the average fold change over three independent biological replicates (n=5). Error bars indicate standard errors of the mean.

Repression of indole metabolism and transcriptional changes of auxin related genes have been reported in Arabidopsis *wat1* mutants and cotton *WATs* silenced plants (Denance et al., 2013; Tang et al., 2019). In our experiments, we monitored the expression of genes involved in auxin transport and auxin responses at different infection time points (**Figure 5**). To evaluate the distribution of auxin in the stems of transgenic plants the expression of three auxin-related genes was studied at different timepoints. Genes *SlPIN1* and *LAX4* were selected on the basis of their function as an exporter and an importer of auxin, respectively (Pattison and Catalá, 2012). *IAA19* is a useful indicator, as it is primarily expressed in the absence of auxin (Tatematsu et al., 2004).

Firstly, we quantified the expression of *SlWAT1*. As expected, the gene was significantly downregulated in RNAi::SlWAT1_1 plants compared to the cv. MM background (**Figures 5A, E**). For the rest of the genes we studied no statistically significant differences were found between cv. MM and transgenic plants (**Figure 5**). At 1 dpi, genes *SlPIN1* and *IAA19* were upregulated in mock inoculated transgenic plants compared to cv.MM. Upon inoculation, their expression was downregulated in RNAi plants (**Figures 5B, D**). The same pattern of expression can be observed for gene *IAA19* also at 7 dpi (**Figure 5H**). At 1 dpi, the expression of the auxin importer *LAX4* was upregulated in RNAi plants inoculated with *Cm* (**Figure 5C**), while at 7 dpi the expression of the gene was comparable to that in cv. MM (**Figure 5G**). In contrast, the expression of auxin efflux transporter *SlPIN1* was upregulated at 7 dpi in infected RNAi plants compared to the cv.MM plants (**Figure 5F**).

**Figure 6:**
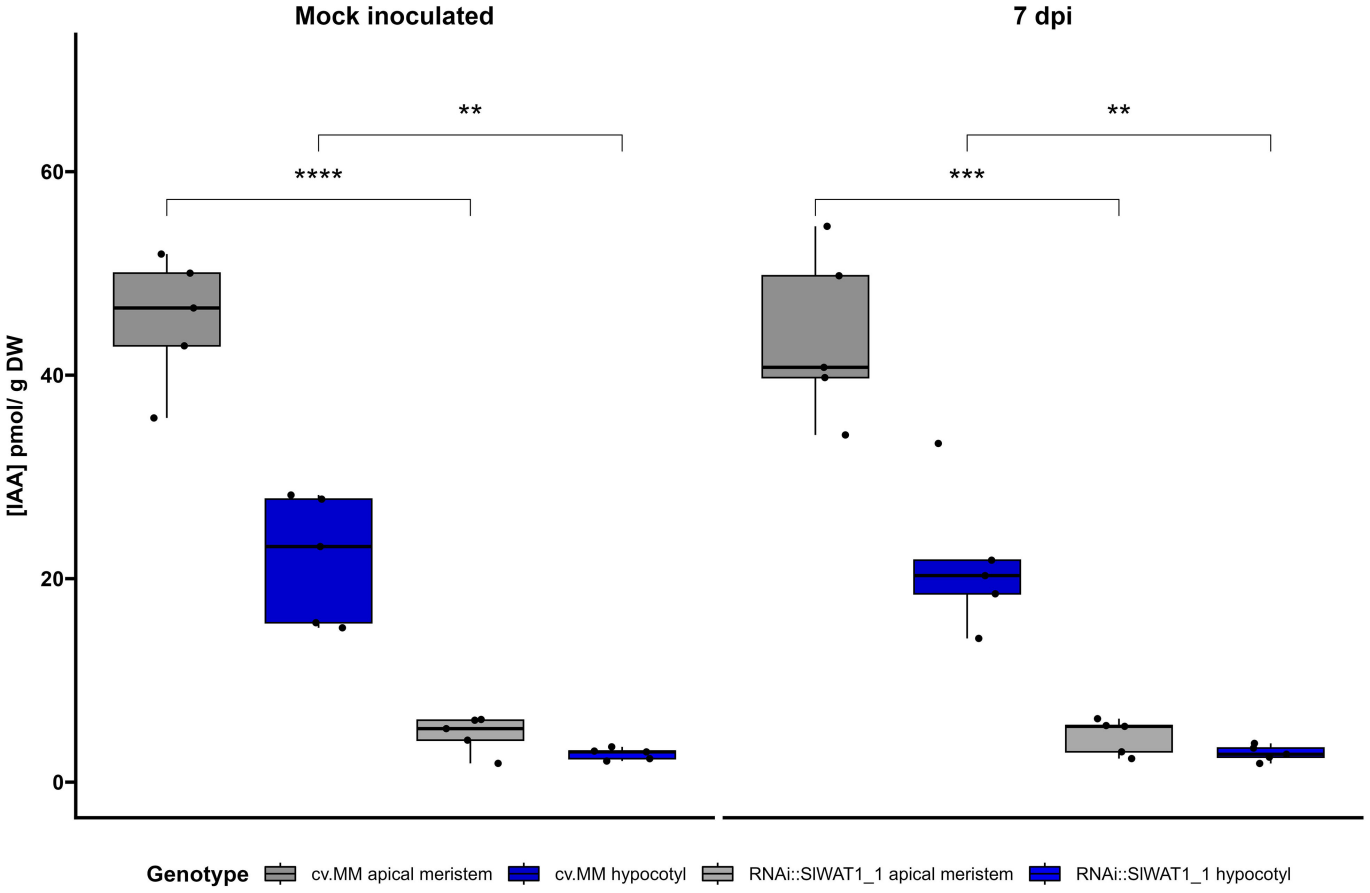
Free IAA content in different stem parts of cv. Moneymaker (cv. MM) and RNAi::SlWAT1_1 plants mock and inoculated at 7 days post inoculation (dpi). Boxplots of IAA concentration (pmol/g DW) in apical meristems and hypocotyls of the two genotypes. Lower and upper box boundaries represent the 25^th^ and 75^th^ percentiles, respectively. Lines in the boxes represent medians of five biological replicates (n=5). (Student’s t-test, **p ≤ 0.01; ***p ≤ 0.001,****p ≤ 0.0001).

Finally, we quantified the levels of auxin in different parts of tomato stems through LC-MS/MS. Free IAA content was quantified at the apical parts of the stem and hypocotyls of RNAi and cv. MM plants that were mock treated or inoculated (7 dpi). Our results confirm that silencing of *SlWAT1* significantly reduces free IAA levels in tomato stems. Consistent with the basipetal auxin transport from source to sink, we also observed a gradient in auxin concentration between the apical meristems and hypocotyls in both genotypes (**Figure 6**).

### Ethylene biosynthesis is downregulated in *SlWAT1* silenced plants

**Figure 7:**
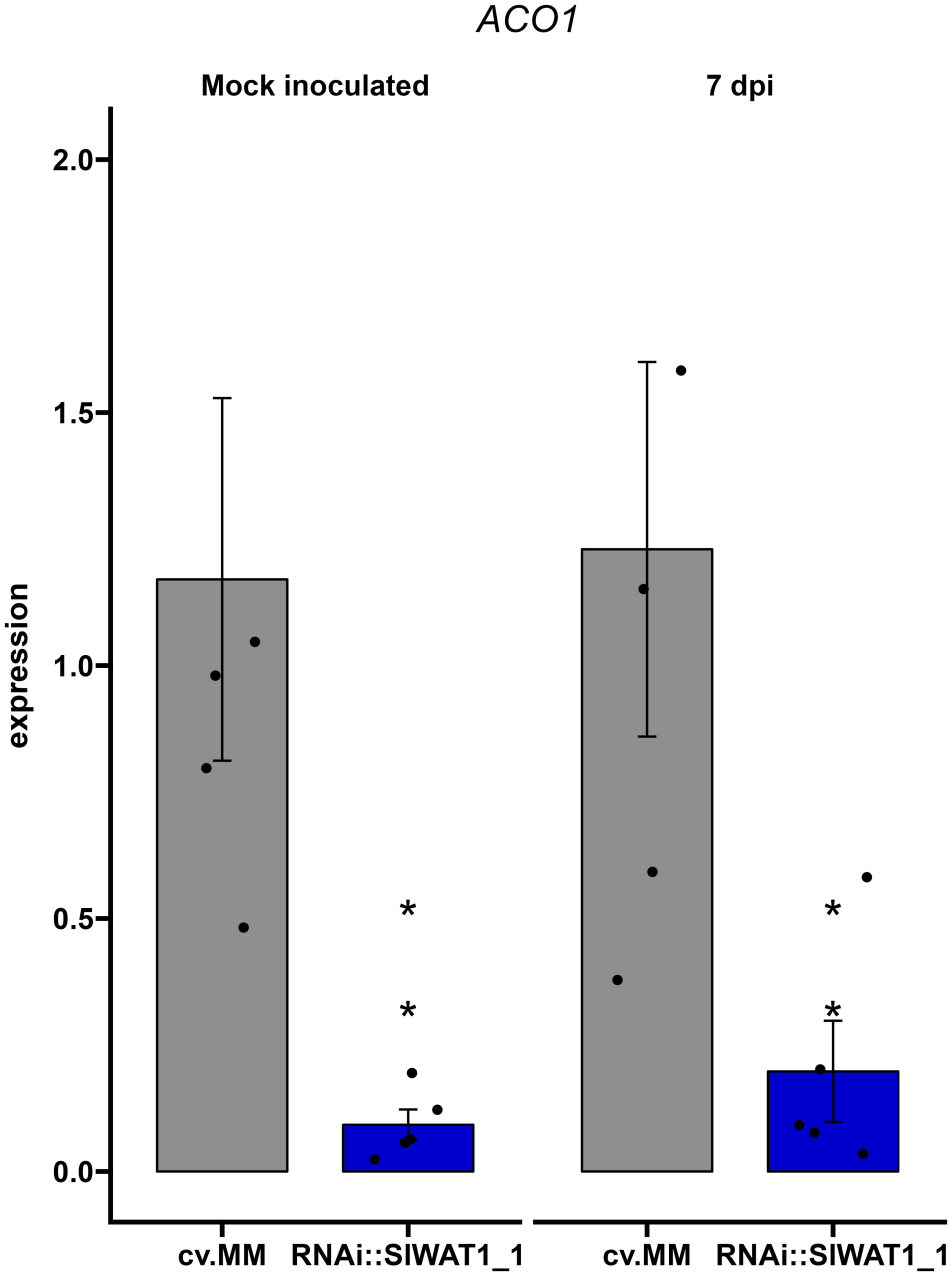
Expression of ethylene biosynthetic gene *ACO1* is constitutively downregulated in RNAi::SlWAT1_1 transgenic plants. Relative expression of gene *ACO1* in mock treated and *Cm* inoculated plants at 7 day post inoculation (dpi). Fold changes were normalized relative to expression of the gene in the control plants of cv. Moneymaker (cv.MM). Bars represent the average fold change over five independent biological replicates (n=5). Error bars indicate standard errors of the mean. Asterisks indicate significant differences to the expression prior to inoculation (Student’s T test, *p≤ 0.05).

Upregulation of ethylene biosynthesis through gene *ACO1* has been shown to contribute to the development of wilting symptoms in *Cm* infected plants (Balaji et al., 2008). Based on our previous observations that silencing of *SlWAT1* reduces symptom development on tomato plants, we hypothesized that silencing of the gene will have an effect on ethylene biosynthesis. Therefore, we examined the expression of gene *ACO1* in the RNAi::SlWAT1_1 plants. We found that the *ACO1* gene is constitutively downregulated in the RNAi plants compared to the cv. MM background, suggesting that ethylene biosynthesis is reduced in the *SlWAT1* silenced tomato plants (**Figures 7**, **S3**).

### Inactivation of *SlWAT1* affects the expression of pathogen virulence factors

**Figure 8:**
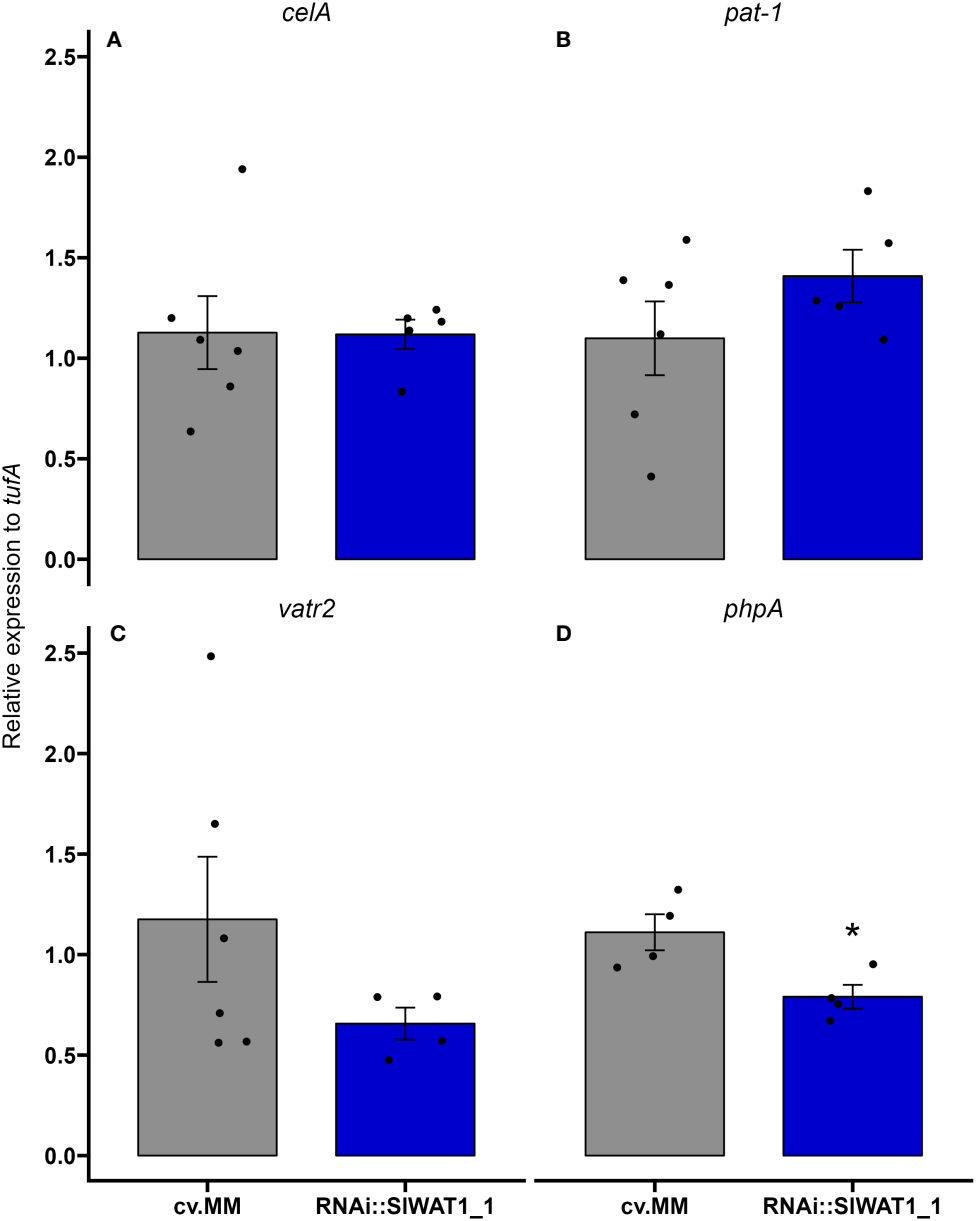
Inactivation of *SlWAT1* affects the expression of specific bacterial virulence genes *in planta.* Relative expression of genes **(A)** *celA*, **(B)** *pat-1*, **(C)** *vatr2* and **(D)** *phpA* on susceptible cv. Moneymaker (cv.MM) and RNAi::SlWAT1_1 plants inoculated with *Cm* strain NCPBB382 at 7 days post inoculation (dpi). Fold changes were normalized relative to expression of the genes in cv.MM plants. Bars represent the average fold change over five independent biological replicates (n=5). Error bars indicate standard errors of the mean. Asterisks indicate significant differences to the expression of the genes in the different genotypes (Student’s T test, *p≤ 0.05).

Recent studies in the interaction between *Pseudomonas syringae* DC3000 and *Arabidopsis* have revealed a role of IAA in the expression of bacterial virulence factors *in planta* and *in vitro* (Kunkel and Harper, 2018). Based on our data that free IAA content in the transgenic plants was significantly lower than in the susceptible cv. MM, we sought to investigate if silencing of *SlWAT1* has an effect on the regulation of *Cm* virulence factors. Plants of cv. MM and RNAi::SlWAT1_1 were inoculated with *Cm* strain NCPBB382 and stems parts were collected at 1 and 7 dpi. Total RNA from infected plants was isolated and was used to monitor the expression of bacterial virulence genes *celA*, *pat-1*, *vatr2* (*virulence associated transcriptional regulator2*) and *phpA*. At 1 dpi, no amplification of bacterial transcripts was possible due to the low proportion of bacterial mRNA in the total isolated RNA. At 7 dpi, no differences were found in the expression of genes *celA* and *pat-1* on cv. MM and RNAi plants (**Figures 8A, B**). Expression of transcription factor *vatr2* and its target *phpA*, however, was found to be downregulated in the RNAi plants at 7 dpi, with *phpA* being significantly downregulated (**Figures 8C, D**). Overall, our results indicate that silencing of *SlWAT1* affects the expression of virulence related genes in *Cm* during bacterial growth *in planta*, possibly through reduced IAA content.

## Discussion

During their co-evolution with plants many pathogens have evolved the ability to manipulate host S genes to establish a compatible interaction (van Schie and Takken, 2014). Loss-of-function of host S genes can possibly alter a compatible interaction into a non-compatible one, leading to pathogen resistance (Pavan et al., 2010; Gawehns et al., 2013; van Schie and Takken, 2014). Here, we report that the loss-of-function of S gene *WAT1* in tomato leads to high tolerance to genetically distinct strains of the bacterial pathogen *Clavibacter michiganensis* (**Figure 1**).

*WAT1* acts as an S gene that enables the infection process of vascular pathogens (Denance et al., 2013; Tang et al., 2019; Hanika et al., 2026). *WAT1* is a tonoplast localized vacuolar auxin transporter, that was first described as a S gene in *Arabidopsis thaliana* (Ranocha et al., 2010). The arabidopsis *wat1-1* mutant was found to be resistant to a broad range of vascular pathogens, including the bacterium *Ralstonia solanacearum* and the fungi *V. dahliae, V. album-altrum* and *F. oxysporum f.* sp. *lycopersici.* Its function as an S gene to fungal vascular wilts has also been reported in cotton and tomato (Tang et al., 2019; Hanika et al., 2026). In this study, we show that inactivation of tomato homolog *SlWAT1* results in strong reduction of symptom development caused by genetically distinct *Cm* strains (**Figure 1**). These findings suggest the function of *WAT1* as an S gene is possibly conserved across plant species and that its loss-of-function can provide broad-spectrum resistance to vascular pathogens. This is an important trait that has been described for several other S genes (van Damme et al., 2008; Acevedo-Garcia et al., 2017; Oliva et al., 2019; Santillán Martínez et al., 2020; Thomazella et al., 2021).

Despite the strong reduction of wilting symptoms, growth of *Cm* was not suppressed by inactivation of *SlWAT1*, in contrast to what it has been reported for other pathogens (Tang et al., 2019; Hanika et al., 2026). According to our initial hypothesis, the residual expression of *SlWAT1* in the RNAi lines was possibly responsible for the mild symptoms observed and the sustained growth of the pathogen. To confirm the results we obtained from the disease assays and to study the effect of a full knock-out in the growth of the pathogen, we included a CRISPR/Cas9 mutant line in our experiments. In accordance with our previous results, we observed strong symptom reduction in *slwat1* mutant tomato plants. Further, we did not detect any significant differences in the *Cm* bacterial titers recovered from the *slwat1* mutants and the susceptible background. As previously observed virulence of Cm does not always correlate with population size. Strain Cmm100, which lacks the two plasmids involved in pathogenicity, cannot cause symptoms on tomato plants and acts as an endophyte. It can, however, grow to population densities that in some cases are higher than the virulent wild type strain. This has been attributed to plant cell death as symptom development advances (Chalupowicz et al., 2010). The high population densities of Cm in SlWAT1 inactivated plants might be an indication of less cell death, which may be a result of downregulation of bacterial virulence factors. As a result of less cell death the pathogen is able to grow to high population densities. This led us to hypothesize that *SlWAT1* is involved in symptom development, rather than sustainment of *Cm*. A major drawback in the use of mutant S genes to gain resistance to pathogens is the possibility of adverse pleiotropy (Sun et al., 2016b), as also observed in the case of tomato *SlWAT1.* Although a full knock-out of the gene led to severe growth defects (**Figure 3**), downregulation of the gene in the RNAi lines resulted in similar tolerance levels to the pathogen without a severe fitness cost on plant growth. In addition to RNAi, alterations in *cis-*regulatory regions of the gene to change its expression (Alonge et al., 2020), might provide a cost-free strategy to gain tolerance to *Cm*. Alternatively, the exploration of allelic variation in tomato germplasm may lead to the identification of natural variants that disrupt the compatible host-pathogen interaction without fitness costs, as it was done in the case of gene *ROD1* in rice (Gao et al., 2021).

Changes in hormonal homeostasis is a common strategy used by pathogens to promote disease. While upregulation of host derived ethylene has been found to promote wilting development by *Cm*, the role of other hormones in the infection process remains unclear (Balaji et al., 2008). Resistance conferred by inactivation of *WAT1* has been associated with altered crosstalk between auxin and SA in Arabidopsis and cotton (Denance et al., 2013; Tang et al., 2019). According to our findings ethylene biosynthesis and auxin content were reduced in *SlWAT1* inactivated plants. We found that the ethylene biosynthetic gene *ACO1*, that is specifically upregulated by *Cm* to promote wilting symptoms was constitutively downregulated in RNAi::SlWAT1_1 plants (**Figures 6**, **S3**). This is also in accordance with previous studies that found that symptom development, but not *Cm* bacterial growth was inhibited on *Nr* ethylene insensitive plants (Balaji et al., 2008).

We also found that the content of free IAA in stem tissues of the RNAi plants was significantly lower than in cv. MM (**Figure 6**). In addition, expression of auxin related genes was altered by *SlWAT1* impaired plants upon *Cm* inoculation (**Figures 5A–H**). At 1 dpi, we found that the expression of auxin influx gene *LAX4* was upregulated in *Cm* inoculated RNAi plants, while the expression of auxin efflux gene *SlPIN1* was upregulated in mock inoculated RNAI plants. Although rather speculative, these changes in the expression of auxin transporter genes might be induced by *Cm* in an attempt to increase auxin influx around the inoculation point. The lower expression of *IAA19* in infected plants (at 1 and 7 dpi) compared to mock inoculated RNAi plants could also suggest that the auxin contents around the inoculation site are indeed increased during infection, since *IAA19* is upregulated in the absence of auxin (Tatematsu et al., 2004). Finally, the upregulation of *SlPIN1* in *Cm* infected *SlWAT1* inactivated plants at 7 dpi, might act as a late compensatory mechanism for the absence of *SlWAT1*, which also facilitates auxin efflux.

Higher contents of SA have been reported for arabidopsis and cotton *WAT1* impaired plants (Denance et al., 2013; Tang et al., 2019). This could be a direct consequence of the reduction of free IAA content, as the SA and auxin hormonal pathways are mutually antagonistic (Wang et al., 2007). Although SA is a known regulator of defenses against pathogens, knowledge on its role in resistance against *Cm* is limited. Recently, it was shown that exogenous application of SA reduces the bacterial populations on tomato cotyledons (Yokotani et al., 2021). The infection of *NahG* transgenic tomato plants with impaired SA accumulation, however, did not result in higher susceptibility to the pathogen (Barda et al., 2015).

Growing evidence suggests that host derived auxin is an important signaling molecule involved in plant-bacteria interactions (Denance et al., 2013; McClerklin et al., 2018; Djami-Tchatchou et al., 2019; Mashiguchi et al., 2019). Recent studies have reported a direct effect of auxin in the regulation of *Pseudomonas syringae DC300* bacterial genes involved in virulence (Aragón et al., 2014; Djami-Tchatchou et al., 2019). Elevated IAA content in Arabidopsis quadruple mutant *tir1 afb1 afb4 afb5*, as well as the addition of IAA in *P. syringae DC300* cultures led to the repression of genes involved in the production of T3SS at early timepoints. The expression of genes involved in late infection stages, however, was significantly upregulated by elevated IAA contents (Djami-Tchatchou et al., 2019). Additionally, auxin produced by bacteria itself can act as a virulence factor (McClerklin et al., 2018). Based on our observations of the significant reduction of symptom development and the significantly lower free IAA content in RNAi::SlWAT1_1 plants, we hypothesized that auxin might play a role in the regulation of *Cm* virulence genes. Therefore, we monitored the transcript levels of virulence factors *celA*, *pat-1*, *vatr2* and *phpA in planta* (**Figure 7**). Interestingly, we observed that transcription factor *vatr2* and its target *phpA* were downregulated at 7 dpi. This suggests that inactivation of *SlWAT1* leads to downregulation of *Cm* virulence genes, possibly through the reduced contents of free IAA in the stems of transgenic plants. Previous, transcriptomics analysis has shown that the virulence factors *celA* and *pat-1* reach the peak of their expression between 24 and 72 hours post inoculation and gene expression is reduced after that point (Chalupowicz et al., 2010). This might be the reason why we did not detect a difference in the expression of *celA* and *pat-1* isolated from cv.MM and RNAi::SlWAT1_1 plants at 7 dpi. To definitely conclude, however, that auxin directly affects the expression of bacterial genes after supplementation of cultures with IAA could be monitored. Moreover, meta-transcriptomics analysis through RNA-seq on infected *SlWAT1* inactivated plants and their susceptible background could be deployed in different experimental timepoints, in order to elucidate the complete pathways involved in the molecular interaction of the organisms (Guo et al., 2021). Future studies on how *Cm* responds to IAA, as well as the production of IAA non-responsive *Cm* mutants, could allow us to fully study and understand the role of auxin as a signaling molecule in the pathosystem. Finally, the possibility of changes in the metabolome of the plants due to perturbations in hormonal changes, might influence the expression of bacterial virulence factors, requires further examination.

## Materials and methods

### Plant materials

The present study included the susceptible *Solanum lycopersicum* cv. Moneymaker MM as a control, T_3_ progeny of two independent stable transformants (RNAi::SlWAT1_1, RNAi::SlWAT1_2) in which the *SlWAT1* gene was silenced through RNAi in cv. MM background and T_2_ progeny of a bi-allelic heterozygous CRISPR/Cas9 generated *slwat1* mutant line (Hanika et al., 2026). Prior to infection, transgenic plantlets were screened for the presence of the RNAi silencing construct based on the presence of the 35S and NPTII markers. *slwat1* mutants were screened for the presence of mutant alleles through PCR based genotyping and sequencing.

Plants were grown in a climate regulated greenhouse compartment at 24°C/18°C under a 12h/12h day/night regime. Relative humidity in the compartment was kept to ~60%.

### DNA isolation and genotyping

For the genotyping of the RNAi transgenic and *slwat1* mutant plants genomic DNA was isolated using a modified protocol for cetyl trimethylammonium bromide (CTAB) extraction method (Doyle, 1991). PCR was performed with DreamTaq DNA polymerase (Thermo Scientific) and target specific primers (**Table S1**). The PCR products of the RNAi transgenic plants were visualized on 1% agarose gel for the screening of the presence of NPTII and 35S transgene markers. PCR products of mutant plants were sequenced through Illumina sequencing (Macrogen Europe, Amsterdam).

### Bacterial strains and growth conditions

Four genetically diverse *Cm* strains were used in the experiments, i.e. *Cm* strains NCPBB382, IPO3356 (rifampicin resistant mutant), CFBP2493 and CFBP5843 were used in the experiments (Jacques et al., 2012). Prior to plant inoculation the strains were grown for two days at 25° C on TBY plates (10 gL^-1^ tryptone, 5 gL^-1^ yeast extract, 5 gL^-1^ sodium chloride, 15 gL^-1^ bacteriological agar). Plates were supplemented with appropriate antibiotics when needed (25 μl/mL rifampicin).

### Disease assay

Tomato plants at the fourth true leaf stage were inoculated by a petiole clipping off method. The petioles of the first two fully expanded leaves were clipped off with razor blades immersed in the bacterial inoculum and 5 μl of the bacterial inoculum were directly pipetted on the lowest wound. Bacterial inocula of the four bacterial strains were prepared by re-suspending cells in Ringer’s buffer to a final concentration of ~10^8^ cfu/ml (OD_600 =_ 0.1). Prior to re-suspension the *Cm* strains were streaked on TBY plates, supplemented with appropriate antibiotics when needed, and incubated at 25°C for two days. Symptom development (wilting) was monitored up to 20 days post inoculation (dpi). A disease index (DI) scale based on the development of wilting symptoms on the leaves was used (0; no symptoms- 5; all leaves wilting). Per strain, 12 transgenic T_3_ plants and three susceptible cv. MM control plants were used. The same procedure was used for the disease assays of *slwat1* mutants. At least five biological replicates of *slwat1* mutants were used in the experiments. Four biological replicates of the susceptible cv. MM were used as controls.

### Bacterial quantification

Bacterial quantification was done through serial dilution plating. Whole stems collected ~1 cm above the lowest inoculation point and up to the apical meristem were sampled at three time points; 4 dpi, 7 dpi and 14 dpi. Stems were pulverized and homogenized in Ringer’s solution (Sigma Aldrich). 50 μl of serial dilutions of the homogenate (10^1^ –10^6^) were plated on SCM-F selective plates (Duchefa Biochemie). The medium was supplemented with 1.9 g L^-1^ yeast extract, 20 μL L^-1^ nalidixic acid (100 mg mL^-1^), 8 mL L^-1^ trimetroprim in MetOH 100% (10 mg/mL), 1 ml L^-1^ cyclohexamide in MetOH 100% (100 mg mL^-1)^, 1 mL potassium tellurite (1%), 50 ml L^-1^ nicotinic acid (2 mg/mL). Plates were supplemented with appropriate antibiotics when necessary (25 μl/mL rifampicin). Plates were incubated at 25°C for 7 days. Colonies on the plates were counted 7 days post plating and the log_10_(cfu+1/g fresh tissue) per plate was calculated. Five biological replicates of RNAi::SlWAT1_1 plants and the susceptible cv. MM were used per time point. Two technical replicates per sample were plated. The same procedure was used for the quantification of *in planta* bacterial titers of strain NCPBB382 in *slwat1* mutants. Five biological replicates per time point were used.

### RNA extraction/cDNA synthesis

Stem samples of ~2 cm in length were collected above the inoculation point. The stems were processed using a Precellys Evolution tissue homogenizer (Bertin Technologies) at 7000 RPM for two rounds of 15 sec, with the cryolysis option on. RNA extraction and on column DNase treatment were done using the RNeasy Mini Kit (Qiagen) and RNase-Free DNase Set (Qiagen) following the manufacturer’s instructions. 500 ng of first strand cDNA was synthesized using the iScript cDNA synthesis kit (Bio-rad).

### Gene expression analysis

Expression levels of tomato genes *SlWAT1*, *ACO1*, *IAA19*, *SlPIN1*, *LAX4* on mock treated and *Cm* infected control and RNAi::SlWAT1_1 transgenic plants were monitored through RT-qPCR at 1 and 7 dpi using specific gene primers (**Table S1**). The expression of bacterial virulence genes *celA*, *pat-1, vatr2* and *phpA* on infected cv. MM and RNAi::SlWAT1_1 was also assessed at 1 and 7 dpi using target specific primers (**Table S1**). 5 and 25 ng of cDNA were used as a template for the reactions for the quantification of plant and bacterial transcripts, respectively. Reactions were done in duplicates. At least four biological samples were used per treatment and time point. RT-qPCR was done on a CFX96 Touch Deep Well Real Time PCR Detection system (Bio-Rad).

Prior to cDNA synthesis for the expression analysis of the bacterial virulence genes, the total RNA isolated was run on 1% agarose gel to confirm the absence of gDNA from the samples. The Livak 2^-ΔΔCt^ method was used to normalize and calibrate transcript values relative to the endogenous *SlEf1α* for tomato and gene *tufA* for the bacterial genes.

### Auxin quantification

Auxin was quantified through Liquid Chromatography- Mass Spectrometry (LC-MS/MS). Plant stem parts were collected and flash frozen in liquid nitrogen. The collected tissue was processed using a Precellys Evolution tissue homogenizer (Bertin Technologies) at 7000 RPM for two rounds of 15 sec, with the cryolysis option on. ~25 mg of tissue were used for the auxin extraction. Ground stem samples were extracted with 1 mL of cold methanol containing [phenyl ^13^C_6_]-IAA (0.1 nmol/mL) as an internal standard in a 2-mL eppendorf tube and purified as previously described (Ruyter-Spira et al., 2011; Schiessl et al., 2019). Samples were filtered through a 0.45 μm Minisart SRP4 filter (Sartorius, Goettingen, Germany) and measured on the same day. Auxin was analyzed on a Waters Xevo TQs tandem quadruple mass spectrometer as previously described (Ruyter-Spira et al., 2011; Gühl et al., 2021).

## Data availability statement

The data presented in the study are deposited in the National Center for Biotechnology Information (NCBI) repository, accession numbers OP832215 and OP832216.

## Author contributions

YB, JW, RV, and EK designed the experiments. YB, JW, and RV supervised the work. EK executed the experiments and performed the data analysis. KH generated the transgenic lines and CRISPR/Cas9 mutants. MMN was involved in the initial characterization of the gene. WK performed the auxin quantification. EK drafted the manuscript. JW, RV, and YB critically reviewed and edited the manuscript. All authors contributed to the article and approved the submitted version.
