# Supplementary material for: Inactivation of tomato WAT1 leads to reduced susceptibility to Clavibacter michiganensis through downregulation of bacterial virulence factors
Source: Front Plant Sci. 2023 May 31;14:1082094. doi: 10.3389/fpls.2023.1082094 (PMC10264788; doi:10.3389/fpls.2023.1082094)
Supplement: Supplementary file 1 [file DataSheet_1.docx]

Supplementary Material

##
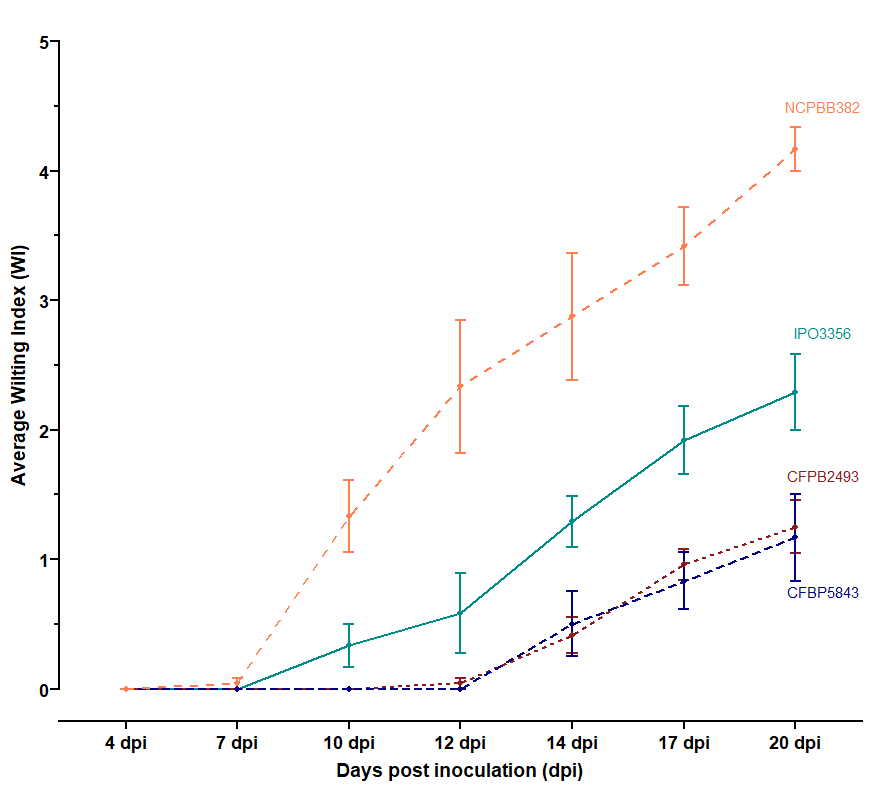


**Supplementary Figure 1**. Strain aggressiveness. Four genetically diverse strain were used for the inoculation of cv.MM plants to assess their aggressiveness. Symptom development was monitored up to 20 dpi. Mean values of three biological replicates (n=3). Bars represent standard errors.


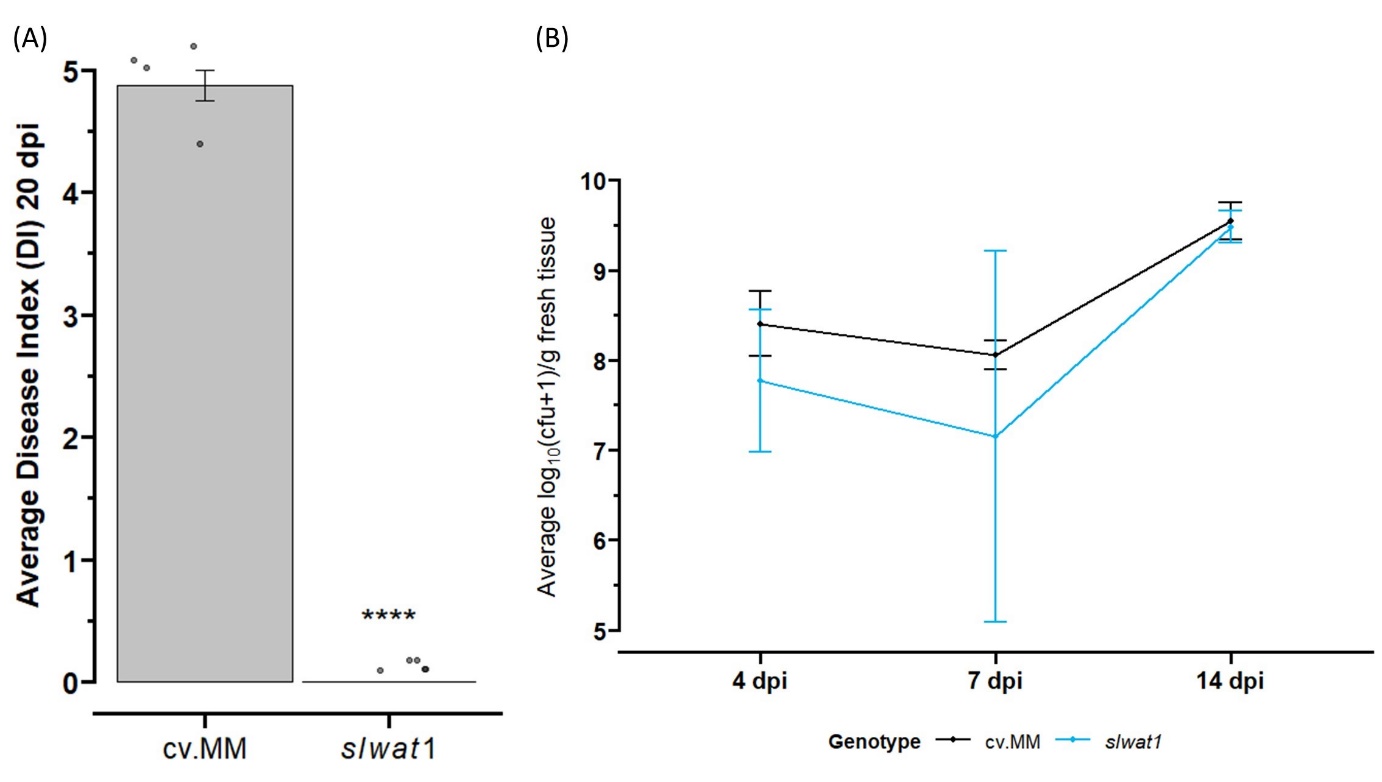


**Supplementary Figure 1**. Symptom development and bacterial dynamics on *slwat1* mutants. (A) Wilting symptom development of slwat1 mutants compared to the background donor susceptible control cv. MM at 20 dpi. Mean values of the slwat1 mutant were significantly different from the cv.MM controls (n=4). (B) *Clavibacter michiganensis* population dynamics in cv.MM and slwat1 mutants. Bacterial titres of bacterial strain NCPBB382 used in the experiments were quantified at 4, 7 and 14 dpi. Three biological replicates (n=3) were used per time point. Lines represent the average log_10_(cfu+1/ g fresh stem tissue) ± stdev. The experiments were repeated independently at least twice with similar results. Asterisks indicate significant differences (Student’s t-test, ****p≤0.0001).


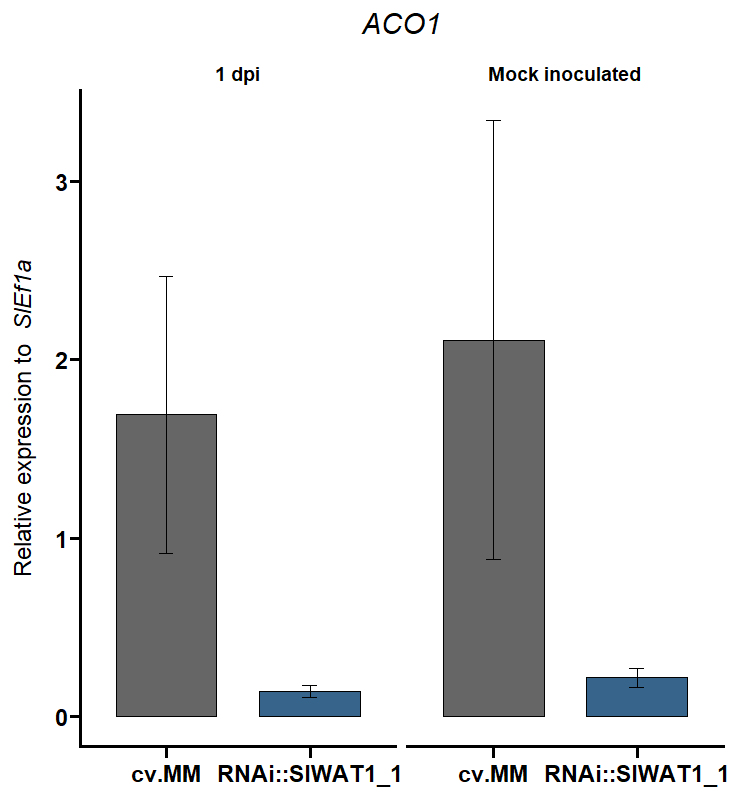


**Supplementary Figure 3.** Expression of ethylene biosynthetic gene. Relative expression of gene *ACO1* in mock treated and *Cm* inoculated plants at 1 dpi. Fold changes were normalised relative to expression of the gene in cv.MM plants. Bars represent the average fold change over five independent biological replicates (n=5). Error bars indicate standard errors of the mean.

**Supplementary Table 1.** List of primers used in this study.

| **Gene** | **Primer sequence (5’-3’)** | **Description** |
| --- | --- | --- |
| *NPTII* | GAAGGGACTGGCTGCTATT | Genotyping of transgenic plants |
|  | AATATCACGGGTAGCCAAC |  |
| *35S* | TACAAAGGCGGCAACAAAC | Genotyping of transgenic plants |
|  | AGCAAGCCTTGAATCGTCC |  |
| *SlWAT1* | CACCGGCCCAACAATTTACAGCCC | Genotyping of mutants |
|  | GAACTAGCCAAGCCTGAGGG |  |
| *SlWAT1* | GGGGGTCCAGTTTTTGTTGC | RT-qPCR |
|  | CTCCGATTATCCCGCCCAAG |  |
| *SlEf1a* | ATTGGAAACGGATATGCCCCT | RT-qPCR |
|  | TCCTTACCTGAACGCCTGTCA |  |
| *ACO1* | ATGGATCGATGTTCCTCCCATG | RT-qPCR |
|  | ATTCGTGTCCCGTCTGTTTG |  |
| *IAA19* | AGTGATCGAAACAGCAGCAG | RT-qPCR |
|  | CCAGAGCAGGCTTTTGACAC |  |
| *SlPIN1* | CCAAGGATCATAGCATGTGG | RT-qPCR |
|  | AGACCAACAGCAATGGAAGC |  |
| *LAX4* | ATGCTGAGAAGCAAGCAGAG | RT-qPCR |
|  | CCAGAGCAGGCTTTTGACAC |  |
| *celA* | CCTCTTCACCACGACTCACC | RT-qPCR |
|  | GCAACGTACATCGGTCTGC |  |
| *pat-1* | TGTCGCGCATAAACAGGATA | RT-qPCR |
|  | AACGAAACACGGGCTATACG |  |
| *vatr2* | GCACATCCTCGAGATCATGG | RT-qPCR |
|  | GTCGATGAAGAAGAGCTTCGTGAC |  |
| *phpA* | CCAATTGCACATGAGTCCAG | RT-qPCR |
|  | GAGTCATCCGTGCCAGTAGC |  |
| *tufA* | CAGGAGCCCGCAGTTCT | RT-qPCR |
|  | GTCCCACCGTCAAGACC |  |
